# Impact of Hysteroscopic Polypectomy on IVF Outcomes in Women with Unexplained Infertility

**DOI:** 10.3390/jcm13164755

**Published:** 2024-08-13

**Authors:** Olga Triantafyllidou, Ilias Korompokis, Stamatia Chasiakou, Panagiotis Bakas, Theodoros Kalampokas, Mara Simopoulou, Despoina Tzanakaki, Emmanouil Kalampokas, Evangelia Panagodimou, Maria Xepapadaki, Panagiotis Christopoulos, Georgios Valsamakis, Nikolaos F Vlahos

**Affiliations:** 1 2nd Department of Obstetrics and Gynaecology, Aretaieio Hospital, National and Kapodistrian University of Athens, Vas. Sofias Str. 76, 11528 Athens, Greece; 2 Hippocratio General Hospital of Athens, Greece V. Sofias 114, 11527 Athens, Greece; 3 Department of Microbiology, General Hospital of Attica “KAT”, Νikis Str. 2, 14561 Athens, Greece

**Keywords:** hysteroscopic polypectomy, in vitro fertilization, clinical pregnancy rate, endometrial polyps, implantation failure

## Abstract

**Objective:** To assess the effect of hysteroscopic polypectomy on the in vitro fertilization (IVF) results in infertile women with at least one prior negative IVF outcome. **Methods:** This retrospective cohort study included women who had attended the “2nd Department of Obstetrics and Gynecology of the National and Kapodistrian University of Athens” and “Iaso” Maternity Hospital from October 2019 to January 2023 for infertility treatment. The medical records of 345 women aged 18–45 years old without abnormal findings in hysterosalpingography (HSG) and with at least one previous failed IVF procedure were analyzed. The male factor was excluded, as well as a prior hysteroscopic removal of polyps. In 67 women, polyps were suspected during initial two-dimensional ultrasound (2D-US) examination. The final sample of the study comprised 40 patients, in which endometrial polyps were removed by hysteroscopy with the use of resectoscope. All patients underwent ovarian stimulation and IVF in the consecutive cycle using a short GnRh antagonist protocol. **Main Results:** After hysteroscopic polypectomy, 29 (72.5%) out of 40 patients had a positive pregnancy result: 26 (65%) clinical and 3 (7.5%) biochemical pregnancies were documented. There was a statistically significant difference between the number of clinical pregnancies before and after polypectomy (*p* < 0.001), as well as between the total number of pregnancies (*p* < 0.001). **Secondary Results**: Women with positive outcome were significantly younger and had significantly lower FSH levels (*p* < 0.007). They also had significantly higher AMH (*p* < 0.009) and peak estradiol levels (*p* < 0.013) and yielded more M II oocytes (*p* < 0.009) and embryos (*p* < 0.002). **Conclusions:** Hysteroscopic polypectomy in women with a suspected endometrial polyp using 2D ultrasound and a history of prior failed IVF attempt improves IVF outcomes in terms of the clinical and total number of pregnancies.

## 1. Introduction

The endometrium is recognized as a dynamic and unique cycling tissue with a distinct and complex mediating role in the implantation mechanism. The role of the endometrium and its underlying mechanisms of embryo implantation remains unclear. The endometrial epithelium plays crucial role during the implantation process, providing correct communication between maternal circulation and the blood vessels of the embryo, and this process should not be interrupted by endometrial pathology [1]. The role of angiogenesis influenced by inflammation and oxidative stress through peroxisome proliferator-activated receptor-gamma has been emphasized [2]. There is a correlation between chronic endometritis and polyps [3]. Endometrial polyps are benign overgrowths of the endometrium containing blood vessels, glands, and stroma [4], affecting about 10% of the general female population [5]. Usually asymptomatic, most polyps are diagnosed during the infertility workup [4]. They can impede with spontaneous fertility and reduce pregnancy rates in assisted reproduction [6]. The exact mechanisms by which endometrial polyps interfere with fertility and impair implantation are poorly understood. It has been suggested that they may be related with mechanical obstruction and space restriction during sperm transportation and embryo implantation, local inflammation, and pathological production of inhibitory factors such as glycodelin, a glycoprotein that inhibits natural killer cell activity and alters the receptivity of the endometrium [4,7,8]. Furthermore, endometrial polyps affect embryo implantation through another mechanism involving decreased HOXA10 and HOXA11 gene expression, which are important molecular markers of endometrial receptivity [4].

In most infertility programs, the uterine cavity is examined using transvaginal ultrasound and hysterosalpingography, while hysteroscopy offers simultaneous diagnosis and treatment of suspected intrauterine abnormalities [9,10]. Hysteroscopy is a simple, low risk, and cost-effective outpatient procedure with an increased success rate [11]. Previous studies suggest that diagnostic hysteroscopy in subfertile women undergoing assisted reproduction can have a beneficial effect on subsequent fertility [12,13,14]. However, only a few studies have compared the pregnancy rates before and after hysteroscopic polypectomy and in vitro fertilization (IVF) in couples with no other fertility issues.

The aim of the present study is to retrospectively assess the impact of hysteroscopic polypectomy on the IVF outcome in infertile women who had already undergone at least one IVF procedure with a negative result. 

## 2. Materials and Methods

This retrospective cohort study included patients who had attended the “2nd Department of Obstetrics and Gynecology of the National and Kapodistrian University of Athens” and “Iaso” Maternity Hospital from October 2019 to January 2023 for infertility treatment. The medical files of 345 women that addressed these two institutions for fertility treatment were examined. The following inclusion criteria had to be met: patient age 18 to 43 years old, absence of male factor infertility, no abnormal findings in hysterosalpingography, at least one failed previous IVF procedure, Follicle Stimulating Hormone (FSH) levels < 16 mIU/mL, and estradiol levels < 60 pg/mL. Exclusion criteria were the following: prior hysteroscopic removal of polyps, FSH levels > 16 mIU/mL, and estradiol levels > 60 pg/mL. In 67 women, polyps were suspected at the initial 2D-US examination. Of these, 15 patients were excluded because they did not meet the study criteria. In 52 women, diagnostic hysteroscopy was performed using a rigid operative hysteroscope. Three patients were diagnosed with small submucosal fibroids < 1 cm, which were removed with the use of the resectoscope, while nine women did not present any uterine abnormality. The final sample of the study comprised 40 patients in which endometrial polyps were detected, and after the hysteroscopic removal of the polyps using a resectoscope, they underwent ovarian stimulation in the consecutive cycle and IVF using the short GnRh antagonist protocol. All of the women signed informed consent forms, and the study was approved by the institutional review committee (Appendix A, flow diagram of the selection process).

## 3. Treatment Schedule

- A fixed r-FSH antagonist protocol was administrated to the participants.
- Serum Anti-Mullerian Hormone (AMH) was measured prior to stimulation.
- Serum level of estradiol and follicle-stimulating hormone (FSH) were measured on the third day of their menstrual cycle.
- Ovarian stimulation was initiated on the same day with 300 IU of recombinant human Follicle-Stimulating Hormone (r-hFSH-alfa) (Gonal-f; MerckSerono Hellas AE) after performing a transvaginal ultrasound in case serum FSH levels were less than 16 mIU/mL and estradiol levels were less than 60 pg/mL.
- A GnRH antagonist, cetrorelix acetate 0.25 mg/day (Cetrotide; Merck Serono Hellas AE), was administrated when a dominant follicle reached a diameter of 14 mm.
- Final oocyte maturation was triggered with 250 mcg of choriogonadotropin alpha (Ovitrelle: Merck Serono Hellas AE) when at least two follicles reached a diameter of 18–20 mm.
- Oocyte Pick Up (OPU) was carried out 34–36 h later.
- Sonographic guided embryo transfer (ET) was performed on day 5 after OPU.
- Serum β-Hcg measurements were performed on day 12 after the OPU and 14 days later clinical pregnancy was confirmed using transvaginal ultrasound.

## 4. Outcome Measures

Clinical pregnancy rate (CPR) was the primary outcome. Secondary outcomes were biochemical pregnancy rate (BPR), as well as the correlation between positive pregnancy outcome and age, hormonal profile, number of mature (metaphase II) oocytes retrieved, and number of embryos. 

## 5. Statistical Analysis

The statistical analysis was performed with a commercially available program (IBM SPSS Statistics for Windows, Version 22.0 IBM Corp. Released 2013. Armonk, NY, USA: IBM Corp.). An a priori power calculation test was performed in order to compute the required sample size of patients in matched/dependent pairs, two-tailed, given a type a error probability of 0.05 and power (type 1-β error probability) of 0.8. The required total sample size was 34 patients (Appendix B). Using a Shapiro–Wilk test, it was found that most categories of numerical data did not follow the normal distribution and are all, therefore, expressed as mean values and interquartile ranges. For the same reason, non-parametric tests were used for the comparison of data between the two groups. The related samples McNemar test was used to compare pregnancy rates before and after the removal of polyps. Also, the independent samples Mann–Whitney U test was used to compare the distribution of clinical data between women with positive and negative pregnancy outcomes. A *p*-value < 0.05 was considered to be statistically significant.

## 6. Results

Forty women with endometrial polyps and previous failed IVF procedures underwent hysteroscopic polypectomy and subsequent IVF, as described previously. The demographics characteristics of all patients are shown in Table 1, and for each variable the mean, range, interquartile range (IQR), first (Q1), and third (Q3) quartiles are listed.

After hysteroscopic polypectomy, 29 (72.5%) out of 40 patients had a positive pregnancy result: 26 (65%) clinical and 3 (7.5%) biochemical pregnancies (positive β-hCG with no ultrasound confirmation of the pregnancy) were documented. Using McNemar’s related samples test, there was a statistically significant difference between the number of clinical pregnancies before and after polypectomy (*p* < 0.001) as well as between the total number of pregnancies (*p* < 0.001). In contrast, there was no statistical significance in biochemical pregnancy rates before and after polypectomy (*p* = 0.250) (Table 2).

Appendix C Pie chart of pregnancy rates after polypectomy and IVF.

Interestingly, when comparing patients according to pregnancy outcome after polypectomy, using the independent samples Mann–Whitney U test, there were statistically significant differences in the distribution of all clinical, hormonal, and embryological data in patients who had positive versus negative pregnancy outcome, as is shown in Table 3. When compared to women with negative pregnancy outcome, women with positive outcome were significantly younger and had significantly lower FSH and day 2 estradiol levels (*p* < 0.05). They also had significantly higher AMH and peak estradiol levels and yielded more M II oocytes and embryos (*p* < 0.04).

## 7. Discussion

In some cases, the evaluation of couples with implantation failure may be challenging, as one fourth of the cases remains undiagnosed [15]. There are two main factors to be taken into consideration, which include embryo quality and uterine integrity. Currently, predictive embryo quality outcomes have become more standardized with the use of universally accepted grading systems, whereas uterine receptivity is an issue of extensive research and debate. Morphological evaluation of the uterine cavity using hysteroscopy is an important parameter, especially in patients who fail to conceive with good quality embryos. There are various studies that confirm the existence of endometrial polyps using hysteroscopy in asymptomatic subfertile women in a rate of 6–31% with normal uterine cavity on 2D ultrasound [16,17,18].

It has been hypothesized that intrauterine polyps may affect endometrial receptivity, thereby affecting ART outcomes. It has been shown that stanniocalcin-1, a protein negatively affecting implantation, is increased in polyps diagnosed in infertile women [19]. A study by Lass et al. in 1999 was the first that evaluated IVF outcomes in women with endometrial polyps. The authors reported no statistically significant difference in pregnancy rates in women that had undergone hysteroscopic polypectomy compared to those without the procedure [20]. Over the years, several studies demonstrated the positive effect of polypectomy on pregnancy rates preceding IVF [21,22,23] or intrauterine insemination (IUI) [24]. 

In a meta-analysis researching the prognostic value of outpatient or office hysteroscopy before IVF in women with a history of repeated implantation failure, it was concluded that the clinical pregnancy rate (CPR) and live birth rate (LBR) were both significantly higher in patients who underwent hysteroscopy compared with patients that did not (*p* < 0.05) [25].

Recently, Marchand et al. conducted a systematic review and meta-analysis on routine hysteroscopic assessments preceding assisted reproductive technology treatment. It was shown that hysteroscopy improved the CRP (*p* < 0.001). No differences were found between the group that underwent hysteroscopy and the control group in terms of LBR, multiple gestations, the miscarriage rate, and the biochemical pregnancy rate [12]. Similarly, another systematic review and meta-analysis by Carrera Roig et al., investigating the role of routine diagnostic hysteroscopy prior to the first IVF attempt, showed significantly higher CPR in patients that had undergone hysteroscopy compared to those that had not. However, they did not find a statistically significant difference between the two groups in LBR [26].

In a Practice Guideline issued by the Global Community of Hysteroscopy Guidelines Committee in 2021, it is argued that routine polypectomy in subfertile women is not sufficiently backed by evidence. Interestingly, cost-effectiveness analysis seems to be in favor of hysteroscopic removal of polyps in women planning to conceive. However, that was based on limited or inconsistent scientific evidence [11]. Although there have been no randomized controlled trials specifically targeting hysteroscopic polypectomy before IVF, there are several studies in favor of the removal of intrauterine polyps prior to any ART treatment [27]. In 2016, a multicentre randomized controlled trial (inSIGHT) concluded that a routine hysteroscopy does not have a positive effect on livebirth rates in infertile women, with no findings in a transvaginal ultrasound before the first IVF treatment [28]. Currently, in a guideline on unexplained infertility issued in 2023 by ESHRE, it is strongly recommended that if ultrasound evaluation of the uterus does not reveal any findings, no further investigation is needed [29].

Notably, in another 2023 guideline on recurrent implantation failure, ESHRE recommends that in couples with repeated implantation failure and a cumulative chance of implantation/pregnancy of more than 60%, hysteroscopy can be considered to be part of the investigation [30]. This guideline disagrees with the results of the TROPHY multicentre randomized controlled trial published in 2016, which concluded that hysteroscopy before IVF in women with a normal ultrasound and recurrent implantation failure does not have a positive effect on livebirth rate [31].

In the present study, we demonstrated that hysteroscopic polypectomy in women with suspected endometrial polyp by 2D ultrasound and with a history of prior failed IVF attempt, resulted in a statistically significant improvement in the number of CRP (65%) and total number of pregnancies (72.5%). This study showed that women with implantation failure should be evaluated more carefully using 2D ultrasound for uterine abnormalities and that hysteroscopy, consequently, could improve IVF outcomes in this group of patients. These conclusions are in accordance with those of Peitsidis et al., who demonstrated that in women who underwent IVF with donor oocytes and had recurrent implantation failure (RIF), undiagnosed uterine pathology was detected during hysteroscopy [32]. 

The findings of our study supported those of Kalampokas et al., where there was a significantly higher cumulative pregnancy rate in women that hysteroscopic polypectomy was performed prior to (IUI) compared to those who were not submitted to the procedure [24]. The study by Vaduva et al. also demonstrated a higher pregnancy rate after hysteroscopic polypectomy prior to IVF (39.43% vs. 23.53% in control group) [21]. 

A retrospective case–control study by Yang et al. reported the same positive effect of polypectomy on CRP (63% vs. 41% in control group, *p* = 0.009) prior to IVF, suggesting that polypectomy may have a positive effect on uterine receptivity. However, LBR between the two groups was the same. Interestingly, the control group comprised women who were randomly selected and were not tested for the presence of endometrial polyps [23].

Another retrospective case–control study by Kavoussi et al. recorded no statistically significant difference in LBR and CPR when comparing patients with and without hysteroscopic polypectomy (*p* < 0.10). However, they suggested that hysteroscopic polypectomy can restore the uterine receptivity status to a level similar to that of women without endometrial polyps [22]. 

It should be mentioned that in our study, women who achieved pregnancy were significantly younger, had significantly lower FSH and day 2 estradiol levels and significantly higher AMH and peak estradiol levels, as well as a greater number of metaphase II oocytes and number of embryos. Therefore, a negative pregnancy outcome in older women could also be attributed to the decline in fertility rate [33], decline in AMH levels, and increased risk of embryonic aneuploidy after the age of 35 [34].

All the results of this study should be interpreted considering its main limitations, which are the sample size, the study design (retrospective cohort study and not a randomized control trial), and that the data originated from two institutions in the same geographical region. 

## 8. Conclusions

Our study reveals a statistically significant percentage of women with undiagnosed uterine abnormalities prior to their first IVF attempt. Furthermore, performing hysteroscopic polypectomy, especially in younger women in our study group, resulted in a higher percentage of CRP and total number of pregnancies. These findings underscore the importance of a thorough initial evaluation of the uterine cavity in infertile women, as the presence of endometrial polyp may impair the outcome of fertility treatment. In conclusion, taking into consideration the emotional, physical, and financial costs of an unsuccessful ART attempt, hysteroscopy should be considered for all women with unexplained infertility, especially for those with advanced maternal age and prior failed IVF attempts. Further randomized control trials should investigate the effect of uterine polyps in IVF to support the effectiveness of hysteroscopic polypectomy.

## Figures and Tables

**Table 1:** Clinical, hormonal and emryologic data.

	Mean	Range	IQR	Q1	Q3
Age	39.83	13.00	4.00	38.00	42.00
FSH	10.38	8.89	3.87	8.33	12.20
AMH	1.13	2.98	1.19	0.55	1.74
Day 2 E2	44.15	40.00	11.5	37.25	48.75
Peak E2	871.99	2270.65	459.27	530.36	989.63
M II oocytes	4.90	12.00	3.00	3.00	6.00
Embryos	2.38	7.00	2.00	1.00	3.00

**Table 2:** Pregnancy rates before and after polypectomy.

	Before Polypectomy	After Polypectomy	*p*-Value
Clinical Pregnancies	0	26 (65%)	<0.001
Biochemical Pregnancies	0	3 (7.5%)	0.250
Total Number of Pregnancies	0	29 (72.5%)	<0.001

**Table 3:** Mean values of clinical, hormonal and embryologic data in patients according to pregnancy outcome (positive/negative) after polypectomy.

	Positive n = 29	Negative n = 11	*p*-Value
Age	39.17	41.55	0.010
FSH	9.80	11.90	0.007
AMH	1.30	0.66	0.009
Day 2 E2	41.66	50.73	0.004
Peak E2	974.29	602.31	0.013
M II oocytes	5.52	3.27	0.009
Embryos	2.83	1.18	0.002

## Data Availability

The data underlying this article will be shared on reasonable request to the corresponding author.
